# *In vivo* hematopoietic Myc activation directs a transcriptional signature in endothelial cells within the bone marrow microenvironment

**DOI:** 10.18632/oncotarget.5217

**Published:** 2015-08-19

**Authors:** Katharina Franke, Baiba Vilne, Olivia Prazeres da Costa, Martina Rudelius, Christian Peschel, Robert A.J. Oostendorp, Ulrich Keller

**Affiliations:** ^1^ III. Medical Department, Technische Universität München, Munich, Germany; ^2^ German Heart Center Munich, Experiential Cardiology, Technische Universität München, Munich, Germany; ^3^ Institute for Medical Microbiology, Immunology and Hygiene (MIH), Technische Universität München, Munich, Germany; ^4^ Institute of Pathology, Universität Würzburg and Comprehensive Cancer Center Mainfranken, Germany; ^5^ German Cancer Consortium (DKTK) and German Cancer Research Center (DKFZ), Heidelberg, Germany

**Keywords:** Myc, leukemia, microenvironment, endothelial cells

## Abstract

Cancer pathogenesis involves tumor-intrinsic genomic aberrations and tumor-cell extrinsic mechanisms such as failure of immunosurveillance and structural and functional changes in the microenvironment. Using *Myc* as a model oncogene we established a conditional mouse bone marrow transduction/transplantation model where the conditional activation of the oncoprotein Myc expressed in the hematopoietic system could be assessed for influencing the host microenvironment. Constitutive ectopic expression of Myc resulted in rapid onset of a lethal myeloproliferative disorder with a median survival of 21 days. In contrast, brief 4-day Myc activation by means of the estrogen receptor (ER) agonist tamoxifen did not result in gross changes in the percentage/frequency of hematopoietic lineages or hematopoietic stem/progenitor cell (HSPC) subsets, nor did Myc activation significantly change the composition of the non-hematopoietic microenvironment defined by phenotyping for CD31, ALCAM, and Sca-1 expression. Transcriptome analysis of endothelial CD45- Ter119- cells from tamoxifen-treated *MycER* bone marrow graft recipients revealed a gene expression signature characterized by specific changes in the Rho subfamily pathway members, in the transcription-translation-machinery and in angiogenesis. In conclusion, intra-hematopoietic Myc activation results in significant transcriptome alterations that can be attributed to oncogene-induced signals from hematopoietic cells towards the microenvironment, e. g. endothelial cells, supporting the idea that even pre-leukemic HSPC highjack components of the niche which then could protect and support the cancer-initiating population.

## INTRODUCTION

Hematopoiesis involves a hierarchy of hematopoietic stem cells (HSC), hematopoietic progenitor cells (HPC), and mature blood cells. Regulation of the cell cycle plays a key role during steady state and challenged blood production and also serves as a checkpoint towards uncontrolled cell proliferation that might lead to blood malignancies. In addition, since the vast majority of HSC are in a quiescent state, the regulation of their entry into the G1 and S phase in response to demand is a crucial step in hematopoiesis. This checkpoint is frequently inactivated in blood cancers and contributes to uncontrolled proliferation of the malignant clone [1-3]. Self-renewal is, for a large part, regulated through extrinsic signals coming from the microenvironment, also termed niche, as a response to the specific requirements of the organisms. E. g., members of the wingless (Wnt) - frizzled (Fzd) pathway play an important role in the regulation of HSC self-renewal and differentiation. Wnt factors activate the canonical pathway through β- and γ-catenin, which in turn results in the up-regulation of target genes like *Myc* [4]. Myc oncoproteins are members of a family of basic region/helix-loop-helix/leucine zipper transcription factors that regulate cell proliferation, differentiation, growth and apoptosis [5, 6]. About 15 % of all genes are regulated by Myc family members [7], and Myc proteins (c-Myc, N-Myc, and L-Myc) are overexpressed in at least 70 % of all aggressive human cancers [8, 9]. c-Myc (Myc) has been shown to play an essential role in regulating the balance between self-renewal and differentiation of HSCs, most probably by altering HSC-microenvironment interactions [10].

Tumors rely not only on genomic aberrations in the tumor cell population but also on an altered microenvironment. The dysregulation of this microenvironment has even been shown to induce a proliferative hematopoietic disorder [11-13]. Extrinsic signals from the microenvironment can, thus, promote malignant transformation of hematopoietic cells. Vice versa, it is also conceivable that early genetic lesions that alone might not suffice to result in malignant transformation could promote shaping of a cancer-supportive niche. This niche might not only promote tumorigenesis, but also could protect and supports cancer cells from therapy [14].

The niche is composed of different cell types which reside in different localizations within the bone marrow (BM). HSCs have been shown to be in direct contact with nestin+ mesenchymal and glial cells [15], (N-cadherin+) osteoblasts (OBC, SNO) [16, 17], CXCL12-abundant reticular (CAR) cells [18], as well as sinusoidal endothelial cells (EC) [19], and endosteal arterioles [20]. Within the niche the anatomical meshwork of these different cell types generates a hypoxic, calcium-rich environment which retains the balance between actively cycling and dormant HSC. Activated HSC are located to perivascular CAR and nestin+ cells near sinusoids. Together with sinusoidal ECs, these cells make up the so-called vascular niche. Multipotent progenitor cells (MPPs) can here enter the circulation [21, 22]. ECs and surrounding perivascular mesenchymal stromal cells (MSCs) promote HSC maintenance by direct contact, as well as by producing secreted factors, such as stromal-derived factor 1α (SDF-1α). Cancer stem cells (CSCs) resemble normal stem cells by occupying these niches and being regulated by the microenvironment to self-renew and differentiate [23]. In addition, tumor cells impair the normal HSC homeostasis, which ultimately, may lead to depletion of normal hematopoiesis [24].

To identify genes and pathways within specific components of the BM microenvironment regulated by oncogenic activity, we chose an *in vivo* model with activatable Myc as a model oncoprotein. Here we show that the constitutive over-expression of Myc in the HSPC compartment results in a myeloproliferative disorder in mice. We further demonstrate that brief Myc activation results in specific transcriptional changes of the microenvironment ECs within the BM.

## RESULTS

### Myc overexpression induces a rapidly lethal myeloproliferative disease

**Figure 1:**
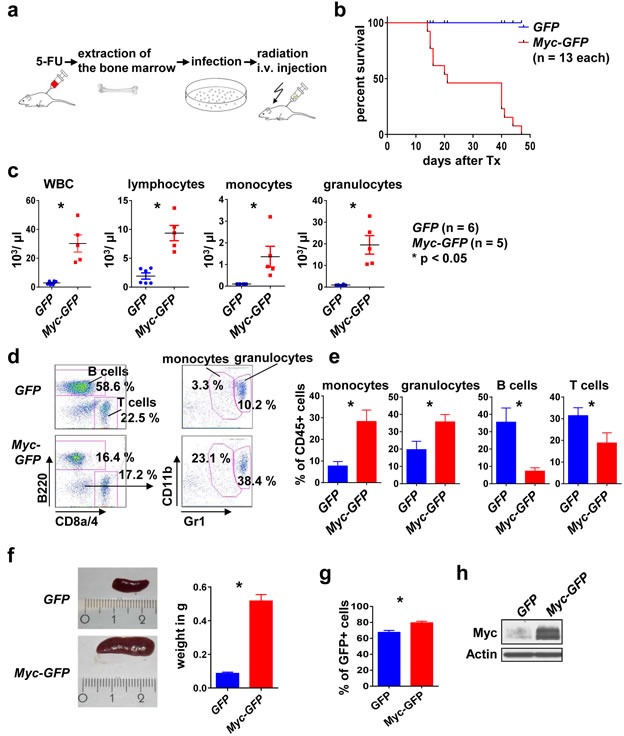
*Myc* overexpression induces a myeloproliferative disease in mice **a.** Scheme of the transplantation model. The BM of the donor mice was mobilized with 5-FU 4 days before the extraction. BM cells were infected *in vitro* with *GFP* or *Myc-GFP* retrovirus. Irradiated recipient mice were transplanted i. v. with infected BM and analyzed after disease onset. **b.** Mice transplanted with *Myc-GFP* infected BM die earlier than the *GFP* controls. Survival curve of the indicated genotypes. **c.** WBC, lymphocytes, monocytes and granulocytes peripheral blood counts of *Myc-GFP* and *GFP* mice at onset of disease. Shown are means ± SEM. * indicates *p* < 0.05. **d.** Representative flow cytometry analysis of the peripheral blood for the indicated surface markers, gated on CD45+ and GFP+ cells. **e.** Quantification of monocytes, granulocytes, B and T cell frequency in the peripheral blood. Shown are means ± SEM. * indicates *p* < 0.05. **f.** Left: Representative images of spleens from a *GFP* control and a *Myc GFP* mouse. Right: quantification. Values are mean ± SEM. * indicates *p* < 0.05. **g.** Percent of GFP+ cells in spleens of the indicated genotypes at disease onset. **h.** Representative immunoblot for Myc expression in the indicated genotypes. Abbreviations: i.v., intravenous; BM, bone marrow; Tx, transplantation; WBC, white blood cell.

Ectopic Myc expression was shown earlier to suffice to induce a myeloid disorder with features of myeloproliferation/acute myeloid leukemia [25]. We hypothesized that a Myc-driven hematopoietic cancer/pre-cancer model could serve as a tool for investigating changes in the microenvironment that could be induced by the cancer cell-intrinsic oncoprotein. For this purpose we aimed to establish a *Myc* leukemia/myeloproliferation model (Figure 1a). We infected BM cells from 5-FU treated donor mice with retrovirus encoding Myc under control of a constitutively active promoter (*Myc-GFP*) or with the empty vector control (*GFP*). The transplanted BM effectively reconstituted the hematopoietic system (data not shown). Transplantation of syngeneic recipient mice with *Myc-GFP* BM resulted in a rapid onset lethal leukemia/myeloproliferation with a median latency of 21 days, while none of the *GFP* control mice died during the observation period (Figure 1b). The Myc-induced disease was characterized by massive leukocytosis, in particular monocyte and granulocyte elevation in the peripheral blood and in the BM (Figure 1c-1e). Furthermore, *Myc-GFP* mice showed massive splenomegaly (Figure 1f), caused by GFP+ cells expressing high levels of Myc (Figure 1g, 1h).

Thus, ectopic Myc expression in a retroviral BM transduction-transplantation model results in a rapid onset myeloproliferative disorder.

### Stable hematopoietic engraftment in mice transplanted with conditional Myc BM

**Figure 2:**
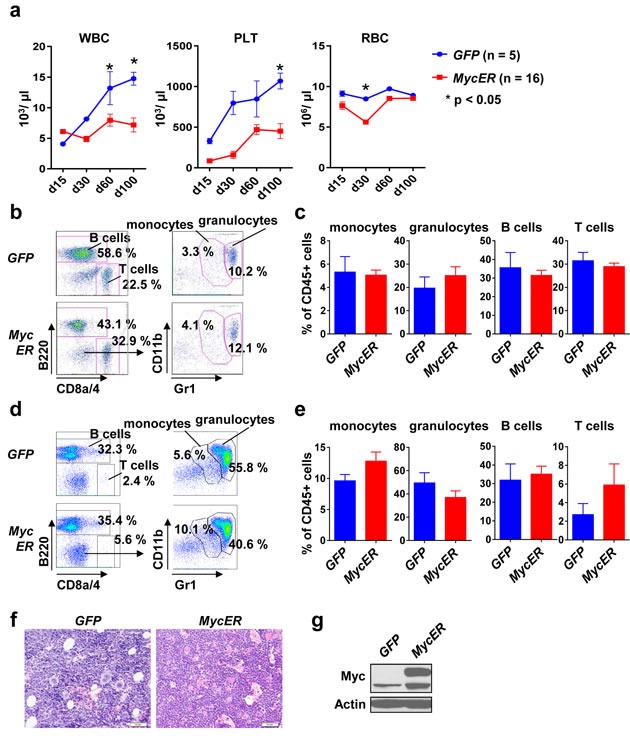
Stable engraftment and phenotype of conditional *MycER* recipient mice **a.** Peripheral blood counts of *GFP* (*n* = 5) and *MycER* (*n* = 16) recipient mice at the indicated time points after transplantation. Values are mean ± SEM. * indicates *p* < 0.05. **b.** Representative flow cytometry analysis for the indicated surface markers. Peripheral blood cells were gated for CD45 and GFP positivity. **c.** Quantification of the indicated peripheral blood cell types 100 days after BM transplantation. Values are mean ± SEM. * indicates *p* < 0.05. **d.** Representative flow cytometry analysis of the BM using the indicated surface markers. Cells were gated for CD45 and GFP positivity. **e.** Quantification of the monocytes, granulocytes, B and T cells in the BM 100 days after transplantation. Values are mean ± SEM. * indicates *p* < 0.05. **f.** BM sections of *GFP* and *MycER* mice using H&E staining. **g.** A representative immunoblot for Myc expression in spleen 100 days after transplantation. Abbreviations: 5-FU, 5-Fluoruracil; WBC, white blood cell; PLT, platelets; RBC, red blood cell; BM, bone marrow.

Severe changes in BM composition as evident in full-blown hematopoietic cancers with massive BM infiltration may hamper detecting subtle cell-cell interactions with the microenvironment. In order to circumvent such a scenario we aimed to establish a conditional *Myc* oncogene model and chose the MycER inducible system [26]. Here, Myc is fused to the synthetic estrogen receptor. The MycER fusion protein is constitutively expressed but largely inactive due to its location within the cytosol by binding to heat shock proteins. Activation of Myc's transcriptional activity is achieved by administration of tamoxifen, which results in Myc translocation into the nucleus [27]. *MycER-GFP* (further referred to as *MycER*) chimeric mice were, again, generated using the retroviral transduction-transplantation model. Transplanted mice showed stable engraftment assessed in the peripheral blood (Figure 2a). *MycER* mice revealed lower peripheral blood counts compared to *GFP* controls, which may have been caused by some leakiness of the ER system, albeit within the normal range (Figure 2a). Importantly, there were no gross disturbances within the leukocyte subsets assessed by flow cytometry, neither in the peripheral blood (Figure 2b, 2c) nor in the BM (Figure 2d, 2e). Furthermore, histopathological analysis of BM section from *MycER* and *GFP* control mice revealed no major differences in cellularity (Figure 2f). In order to evaluate MycER expression we performed immunoblotting from spleen tissue, revealing the expected ectopic MycER expression (Figure 2g).

Our results thus indicate that the mice reconstituted with *MycER*-infected BM show stable engraftment without gross abnormalities and without evidence for myeloproliferation in the absence of tamoxifen.

### *In vivo* Myc activation results in Myc target gene transcription and EC reduction

**Figure 3:**
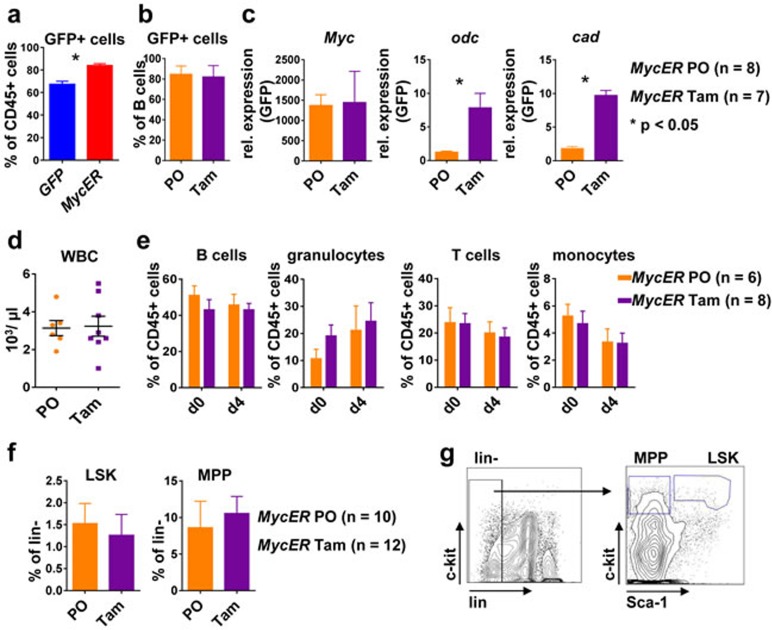
Conditional Myc activation results in target gene expression *in vivo* **a.** Flow cytometry of peripheral blood. Shown is the percentage of GFP+ leukocytes nine weeks after transplantation of BM of the indicated genotype. Values are mean ± SEM. * indicates *p* < 0.05. **b.** Percent of GFP+ B cells in the spleen of *MycER* mice treated with tamoxifen (Tam, *n* = 7) or carrier only (PO, *n* = 8) for 4 days as assessed by flow cytometry. **c.** B220+ splenic B cells isolated from *MycER* mice treated with PO or with Tam were assessed for expression of the indicated transcripts. Shown is the relative expression as compared to *GFP* control transplanted mice. Values are mean ± SEM. * indicates *p* < 0.05. **d.** WBC numbers in the peripheral blood of *MycER* mice treated with PO (*n* = 6) or Tam (*n* = 8) for 4 days. Values are mean ± SEM. **e.** Flow cytometry quantification of the indicated cell types in peripheral blood after the MycER activation. d0: before treatment; d4: after 4 days Tam or PO treatment. Values are mean ± SEM. **f.** Frequency of HSPC subpopulations LSK and MPP in the BM of PO (*n* = 10) or Tam (*n* = 12) treated *MycER* mice. Values are mean ± SEM. **g.** Shown are representative flow cytometry dot plots of BM cells stained for lineage markers (Ter-119, CD11b, B220, Gr-1 and CD3e), c-kit and Sca-1. Abbreviations: Tam, tamoxifen; PO, peanut oil; Odc; ornithin decarboxylase; Cad, carbamoyl-phosphate synthetase 2; WBC, white blood cells; HSPC, hematopoietic stem/progenitor cells; LSK, Lin−Sca-1+c-Kit+; MPP, multipotent progenitor Lin−Sca-1-c-Kit+; BM, bone marrow.

To test MycER activation *in vivo*, nine weeks after transplantation and after a stable reconstitution of high level GFP+ hematopoiesis (Figure 3a), *MycER* mice were treated with tamoxifen (Tam) or carrier (peanut oil, PO) for four days. Analysis of B220+ purified splenic B cells by flow cytometry revealed GFP positivity of 80 % of B cells (Figure 3b). In these cells transcripts for the established Myc target genes *ornithin decarboxylase* (*Odc*) and *carbamoyl-phosphate synthetase 2* (*Cad)* [28, 29] was significantly induced (Figure 3c). Evaluation of the effects of Myc activation on hematopoiesis did not show alterations in peripheral blood cell numbers or in the composition of the leukocyte subgroups (Figure 3d, 3e). Furthermore, we did not find significant alterations in the composition of hematopoietic stem or progenitor subsets upon Tam vs. carrier only treatment (Figure 3f, 3g). In particular, brief Myc activation did not result in leukemic myeloproliferation or in an overt block of the maintenance of HSC or an expansion of MPP that has been reported for prolonged Myc overexpression, although histologically a slight myeloproliferation in the BM could be observed [10].

**Figure 4:**
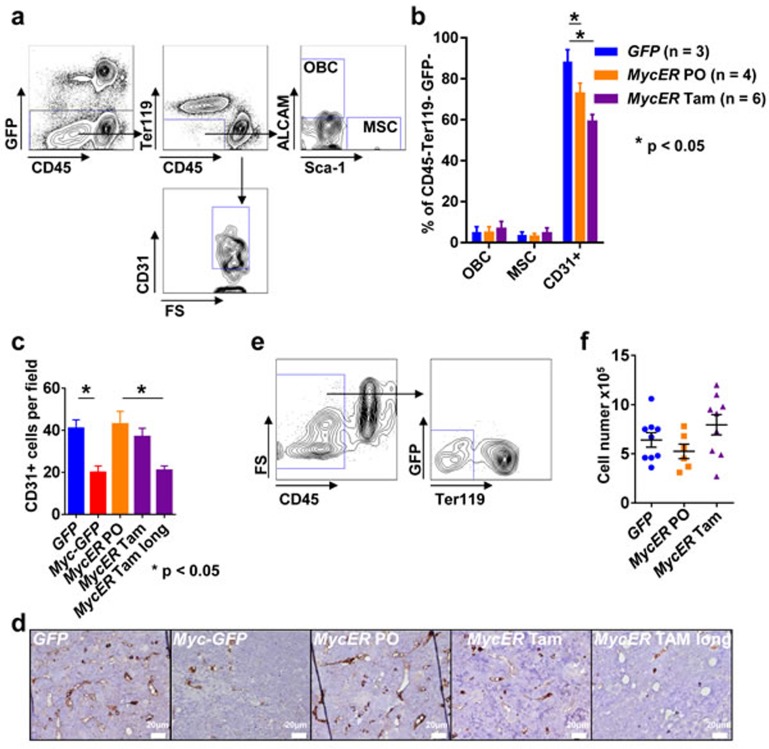
Analysis of the BM microenvironment **a.** Representative flow cytometric analysis of the BM microenvironmental cells nine weeks after transplantation. **b.** Composition of the BM microenvironment: ALCAM+ Sca-1- (OBC), ALCAM-Sca-1+ (MSC) and CD31+ (EC) populations. The graphs represent the mean ± SEM. * indicates *p* < 0.05. (**c**, **d**) BM sections of the indicated genotypes and conditions (*MycER* Tam long indicates tamoxifen treatment until onset of disease; *n* = 3 each) were analyzed for CD31+ cells using immunohistochemistry in three different 200x high power fields. **c.** Mean cell number per 200x high power field ± SEM. * indicates statistically significant differences (*p* < 0.05). **d.** Representative images of the indicated genotypes and conditions. White bars: 20μm. **e.** Representative flow cytometry sorting of CD45- Ter119- GFP-cells for transcriptome analysis. **f.** Total cell number (GFP- CD45- Ter119-) obtained after sorting of 6.7 × 10^5^ cells from the three indicated recipient groups. Values are mean ± SEM. Abbreviations: FS, forward scatter; SS, side scatter; Tam, tamoxifen; PO, peanut oil; MSC, mesenchymal stromal cells; OBC, osteoblast cells; BM, bone marrow.

We next aimed to identify oncoprotein-regulated genes in a pre-leukemic microenvironment in the BM of the established *MycER in vivo* model. For this purpose, since Tam treatment most likely resembles an early pre-leukemic state, we chose to study the vascular compartment which is an immediate compartment to come into contact with the *MycER* hematopoietic cells. In order to particularly study the sinusoidal compartment, we isolated non-hematopoietic BM cells as the CD45- Ter119- GFP-population from flushed BM without collagenase treatment of adjacent endosteal compartments. Using staining with CD31 (PECAM-1), ALCAM (CD166) and Sca-1, we distinguished endothelial cells (ECs), OBCs and MSCs (Figure 4a) [30]. The most abundant population was the CD31+ ALCAM-Sca-1− EC population. In the flushed marrows, the CD31- ALCAM+ Sca-1- OBC population and ALCAM-Sca-1+ population containing MSCs [30] represented the least abundant subpopulations. No significant differences between the *GFP* control mice and the *MycER* mice treated with PO or Tam could be detected with regard to OBC and MSC. The CD31+ ECs were significantly more abundant in the *GFP* control mice compared to MycER/Tam mice (Figure 4b). This EC reduction was a persistent event which could also be seen in moribund *Myc-GFP* and long-term Tam treated *MycER* mice (Figure 4c, 4d).

Collectively our data show that a brief activation of hematopoietic MycER results in the activation of Myc target genes but not in tangible alterations in the hematopoietic compartment or in the compartment of MSC and OBC, but showed a reduction of the EC compartment.

### Myc directs transcriptional changes in EC of the BM environment

**Figure 5:**
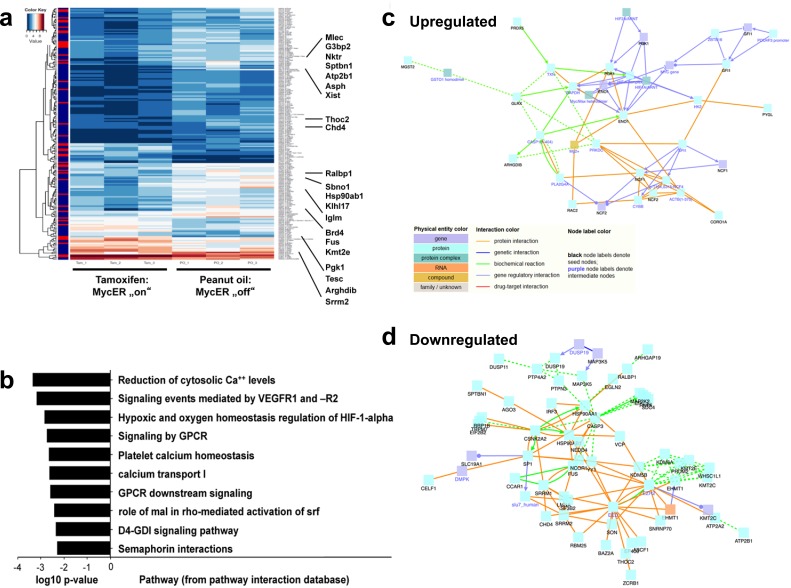
Myc directs transcriptional changes in EC within the BM microenvironment **a.** Heatmap image of genes that are significant (*p* < 0.05) differentially (log2 fold change (FC) ≥ 1.0 or ≤ −1.0) expressed (DEGs) in the non-hematopoietic EC BM cells of *MycER* mice treated with PO versus Tam. Highlighted are the 21 most regulated genes. For a complete list of analyzed genes, see supplemental Tables S1 and S2. **b.** Pathway enrichment of the DEGs (Tables S1 and S2), **c.** Network of the up-regulated DEGs: protein-protein interaction (PPI) information was visualized using the “induced networks module” tool from the ConsensusPathDB database (http://consensuspathdb.org/) using only high-confidence binary interactions and an intermediate nodes z-score threshold of 10. **d.** Similar network analysis, but now showing down-regulated DEGs, using an intermediate nodes z-score threshold of 40.

To determine how *MycER* cells from Tam treated mice (Figure 3c) affect ECs (GFP- CD31+ CD45- Ter119-) as compared to PO control mice, we sorted ECs (Figure 4a-4f), and performed transcriptome analysis. Genes that were significant (*p* < 0.05) differentially (log2 fold change (FC) ≥ 1.0 or ≤ −1.0) expressed (DEGs) in ECs of *MycER* mice treated with Tam or PO are shown in Figure 5a (see also supplemental Table S1 and Table S2). Thirty four of these differently expressed Myc-regulated genes were up-regulated in the Tam treated cohort, while 194 genes were down-regulated. Hierarchical clustering of these DEGs showed clear separation of the PO- and TAM-treated *MycER* animals (Figure 5a). In enrichment analyses performed to highlight the over-represented categories in pathways [31] (Figure 5b, supplemental Table S3), the DEGs revealed that Myc activation (*MycER* cells, Tam-treated) up-regulates hypoxia signaling and glucose metabolism (Figure 5c, Table S3, ordered by significance level), while down-regulating VEGFR, calcium clearance, small GTPase RhoB signaling pathways (Figure 5d). These analyses further revealed that transcription-translation processes, the histone demethylation machinery, and angiogenesis were significantly regulated in the endothelial compartment. Network analysis revealed protein interactions between the DEGs with prominent up-regulated number of connections to Eno1 and Pgk1, and down-regulated connections to Csnk2a2 and polycomb complex genes (Figure 5c, 5d, respectively).

## DISCUSSION

It is well-established that cancers rely not only on genetic disorders within the tumor cell population but also on changes in the microenvironment of the tumor. To find regulated genes in the microenvironment of an *in vivo* model we chose *Myc* as a model oncogene which could be conditionally expressed in HSPCs. The permanent overexpression of Myc resulted in the rapid onset of a myeloproliferative disease in mice with splenomegaly. We demonstrated that the mice transplanted with conditional *MycER*-infected BM showed a stable engraftment without significant changes in comparison to the *GFP* control mice. Also no evidence for leukemic myeloproliferation could be seen in the *MycER* mice demonstrating that the system was to a larger extent leaky.

The study of pre-leukemic/pre-cancerous changes is challenging. The conditional *MycER* model allows for brief activation of Myc which results in transcription of the reported Myc target genes *Odc* and *Cad*. Importantly, after brief Myc activation overt myeloproliferation was not detectable. Others have shown that the overexpression of Myc in the HSPC compartment results in a block of HSC self-renewal and an expansion of the MPP [10], which we also did not observe in the *MycER* mice treated with Tam or carrier solution. Prolonged Tam treatment induced apoptosis in the MPP compartment and enhanced proliferation in the HSC [32]. The four day treatment with Tam we performed had however no significant effect on these HSPC populations. Thus, brief conditional activation of Myc does not alter the hematopoietic system in the BM, which renders this model suitable to study pre-leukemic changes of the BM microenvironment without disturbing effects caused by malignant transformations.

To study oncoprotein-induced changes within the BM microenvironment we more closely investigated the EC-enriched CD45- Ter119- GFP- non-hematopoietic host population. Our experiments revealed that *MycER* Tam-activated hematopoietic cells reduced the number of ECs in the BM. ECs play multiple roles within the BM. They secrete SDF-1α (CXCL12), which restrains the HSPCs in the BM microenvironment through the CXCR4-CXCL12 signaling pathway [21-23]. Secondly, the fenestrated endothelium allows HPCs to enter the blood stream and vice versa. In addition, the sinusoidal endothelial compartment and its surrounding Lepr+ nestin^dim^ NG2^−^ perivascular cells have been shown to maintain actively cycling HSCs through secretion of stem cell factor (SCF) [33]. More quiescent HSCs are located closer to ECs surrounded by Nestin+ NG2+ cells [34]. The reduction of EC in pre-leukemic mice may be a first change leading to a hijacked microenvironment, i.e. a tumor cell-driven environment supporting survival and proliferation of tumor cells [14]. Our observations indicate that pre-malignant cells transform the BM microenvironment very early during leukemogenesis. Pathway enrichment analysis of the DEGs showed that several different pathways are involved in those early changes in the transcriptome of ECs. In particular the Rho subfamily pathways, the transcription-translation machinery, the angiogenesis, and oxygen homeostasis pathways are affected by the presence of Myc^high^ cells. Although increased angiogenesis is a hallmark of cancer [35], the early pre-leukemic changes are characterized by reduced number of EC, reduced VEGFR1, and -2 signaling, and increased expression of the HIF-1α target genes *Pgk1*- and *Eno1* by CD31+ CD45- Ter119- cells. As VEGFR signaling induces proliferation, survival and migration of EC [36, 37], it is likely that inhibition of this pathway also results in a reduced proliferation or increased apoptosis. In addition to these pathways, the decreased expression of the RhoGEF *Arhgef12* (also known as LARG), *Rac2*, *Rhob*, and its effector *Diaph1*, which are involved in stress fiber formation and cellular movement [38, 39], vascular trafficking [40], transcriptional dynamics and cell cycle [41], suggests that pre-leukemic Myc^high^ cells also affect remodeling of the vascular network. Regulation of these early events in leukemic transformation by conditional expression of Myc reveals one crucial point of action, i. e. how evolving cancer cells not only reprogram mesenchymal but also endothelial niche cells.

In summary, our data generated in an *in vivo* conditional oncogene model hint towards pre-leukemic steps in remodeling BM EC numbers, movement and response to hypoxia. Our analyses thus provide evidence for involvement of BM ECs in early pre-leukemic changes in the niche, and provide a rationale for further investigating endothelial targets for niche-directed therapies.

## MATERIALS AND METHODS

### Mice

Mice were kept in microisolators under SPF conditions according to FELASA recommendations. All animal experiments were performed in accordance with FELASA guidelines and the regional animal ethics committee approvals. C57Bl6/J mice were received from Janvier Labs (France).

### Bone marrow infection and transplantation

Recipient mice were lethally irradiated with 8.5 Gy and received donor cells via the tail vein on the next day, as described previously [42]. After 9 weeks, mice were sacrificed, and BM, spleen, and blood cells were analyzed.

To generate *MycER* expressing cells, retrovirus was obtained through transfection of 293-derived ΦNX Eco cells with MSCV-IRES-MycER-GFP [26] using lipofectamine 2000 (Invitrogen, Karlsruhe, Germany). To facilitate transfection of cells, donor mice were pretreated with 150 mg/kg 5-FU (medac, Wedel, Germany) four days prior infection [43]. Transfected donor cells were injected i.v. in lethally irradiated recipient mice. Four weeks after transplantation, each recipient was bled and tested for level of engraftment.

To induce the MycER activation, tamoxifen (Sigma-Aldrich, Taufkirchen, Germany) was dissolved in peanut oil (Sigma-Aldrich, Taufkirchen, Germany) and administered by daily intraperitoneal (IP) injection for four days at a dose of 1 mg/20g/mouse per day. For long-term treatment with tamoxifen mice received a special diet containing 400 mg/kg tamoxifen (Ssniff, Soest, Germany). Since Myc activation is not equally efficient in every single mouse, overexpression of *c-Myc* and the activation of the target genes *Odc* and *Cad* was assessed in all recipient mice. Only mice showing a robust activation of Myc expression with accompanied increase in *Odc* and *Cad* were used as Myc+ recipient mice in our analyses.

### Flow cytometry and cell isolation

Cell suspensions were stained with antibodies for surface antigens anti-Ly-6G (Gr-1)-PE (clone RB6-8C5), anti-CD4-PE-Cy5 (clone GK1.5), anti-CD8a-PE-Cy5 (clone 53-6.7), anti-CD11b-APC-eFluor780 (clone M1/70), anti-CD45R (B220)-PE-Cy7 (clone RA3-6B2), anti-CD45-eFluor450 (clone 30-F11), anti-CD166 (ALCAM)-PE (clone eBio ALC48), anti-Ter-119-eFluor450 (clone TER-119), anti-CD31 (PECAM-1)-APC (clone 390), anti-CD117 (c-Kit)-PE (clone 2B8) and anti-Ly-6A (Sca-1)-PE-Cy7 (clone D7) in 1 × PBS (PAA, Cölbe, Germany), 0.5 % BSA (Roth, Karlsruhe, Germany) buffer for 15 min on ice in the dark. All antibodies are from eBioscience (Frankfurt, Germany). Cells viability was assessed by Propidium iodide (PI) stain (BioLegend, London, United Kingdom). FACS analysis was performed on a CyAn ADP Lx 9 (Beckman Coulter, Krefeld, Germany) flow cytometer. FACS data were analyzed using the FlowJo software (TreeStar Inc).

Isolation of B cells from spleen was achieved by labeling the B cells with an antibody against B220 (CD45R) conjugated to MicroBeads (Miltenyi Biotec, Tokyo, Japan). The not magnetically labeled non-B cells were depleted by retaining them on a MACS^®^ Column (LS column, Miltenyi, Tokyo, Japan) in the magnetic field of a MACS Separator, while the labeled B cells bind the column.

### Tissue and organ samples and cell sorting

Blood samples were collected into 1.2 ml heparinized tubes (Sarstedt AG, Nümbrecht, Germany). Blood cells were counted on a Scil Vet ABC (ScilAnimal Care, Viernheim, Germany). BM cells were flushed from femurs and tibias with HF2+ buffer (Hank's balanced salt solution, supplemented with 2 % FCS, 10 mM HEPES buffer and antibiotics, Invitrogen, Karlsruhe, Germany). Spleens were passed through 70 μm nylon Cell Strainer (BD Biosciences, Heidelberg, Germany). Viable cells were counted using Trypan Blue (Invitrogen, Karlsruhe, Germany) in a Neubauer hemocytometer.

Cell population of the BM were sorted on the MoFlo Legacy 14 color cell sorter (Beckman Coulter, Krefeld, Germany) supplied with Summit 4.3 software (Beckman Coulter). Cell suspensions were stained with antibodies for surface antigens anti-Ter-119-eFluor450 (clone TER-119) and anti-CD45-APC-eFluor780 (clone 30-F11) in 1 × PBS (PAA, Cölbe, Germany), 0.5 % BSA (Roth, Karlsruhe, Germany) buffer for 15 min on ice in the dark. All antibodies are from eBioscience (Frankfurt, Germany). Gated was for the Ter-119 negative, CD45 negative and GFP negative population.

### Real-time PCR and gene expression analysis

Copy-DNA (cDNA) from mouse samples and cultured cells was reverse transcribed using the Omniscript^®^ RT Kit on mRNA isolated with the RNeasy^®^ Mini Kit (both from Qiagen, Hilden, Germany). Real-time PCR was performed using Platinum SYBR-Green Q PCR SuperMix-UDG (Invitrogen, Karlsruhe, Germany) on ABI Prism 7700 (Applied Biosystems, Wien, Austria). Data analysis was done by comparing Ct values with a control sample set as 1 and normalized to the expression of *ubi* (*ubiquitin*). Sequences for primers are available upon request.

### Immunoblotting

Protein extracts (30 μg per lane) were electrophoretically separated on a SDS-PAGE gel, transferred to PVDF-membranes (Millipore, Darmstadt, Germany) and blotted with antibodies specific for c-Myc (Santa Cruz Biotechnology, Heidelberg, Germany) and β-Actin (Sigma-Aldrich, Taufkirchen, Germany).

### Histology and immunohistochemistry

Bones were obtained from the *GFP* and *MycER* mice. Slides of 5- to 6-μm sections cut from formalin-fixed, paraffin-embedded tissues were deparaffinized and stained with hematoxylin and eosin (Dako, Jena, Germany), dehydrated, and then covered with a coverslip. For immunohistochemistry 2-μm sections were deparaffinized. Antigen retrieval was performed by pressure cooking in citrate buffer (*pH* = 6) for 7 minutes. Primary CD31 antibody (DAKO; diluation 1:80) was incubated overnight at 4°C. Antibody detection was performed using the DAKO REAL detection kit (DAKO) accoding to the manufacturer's instructions. High-power fields (200x) of *n* = 3 samples of the indicated genotypes and conditions were analyzed.

### Microarray analysis

Gene expression profiling was performed on Affymetrix Mouse Gene ST 1.0 high-density oligonucleotide arrays. Total cellular RNA was isolated from sorted cells using RNA isolation kit (RNeasy^®^ Mini Kit; Qiagen, Hilden, Germany), and further on labeled, fragmented, and hybridized to the arrays using the WT Expression Kit (Affymetrix). Raw data analysis was performed with R and Bioconductor [44]. Arrays were assessed for quality and RMA-normalized.

For two-way comparisons, the limma [45] t-statistic approach with Benjamini-Hochberg multiple testing correction [46] was used to select the differentially expressed genes (DEGs) from the RMA-normalized gene expression data. Genes were defined as differentially expressed if they had a −1 ≥ log2FC ≥ 1 and nominal *P* ≤ 0.05 (not corrected for multiple testing) between the two conditions being compared. Hierarchical clustering of the differentially expressed genes (DEG) and visualization of the resulting heatmap was done using R heatmap.2 function from the gplots package [44].

Biochemical Pathway information was collected from ConsensusPathDB http://consensuspathdb.org, [31] which is an integrative database currently containing 4,601 Biochemical Pathways in Homo sapiens from 32 public resources such as KEGG [47], Reactome [48], Wikipathways [49] and NetPath [50]. We first collected all Pathways associated with each of the differentially expressed genes (DEGs; −1 ≥ log2FC ≥ 1 and *P* ≤ 0.05, not corrected for multiple testing) genes and performed enrichment analysis to highlight the over-represented categories. Fisher's exact test as implemented in R http://www.r-project.org was used to calculate the statistical significance of the overlaps, thereafter the false discovery rate (FDR) was controlled using the Benjamini-Hochberg (BH) procedure [46].

Protein-protein interaction (PPI) information was taken from ConsensusPathDB http://consensuspathdb.org, [31] which is an integrative interaction database currently containing 215,541 unique interactions from 32 public resources such as IntAct [51], BioGrid [52] HPRD [53] and CORUM [54]. ConsensusPathDB was searched for direct interactions between the differentially expressed genes (DEGs; −1 ≥ log2FC ≥ 1 and *P* ≤ 0.05, not corrected for multiple testing). Network visualization was performed using the induced network modules in the gene set analysis tool of ConsensusPathDB.

### Statistical analysis

Statistical analyses were performed using the statistical functions of Excel (Microsoft, Hamburg, Germany) and GraphPad Prism (GraphPad Software, San Diego, California, USA). The bars shown represent the standard error of the mean (SEM). *T*-tests were used to analyze statistical differences between two groups. When comparing ≥ 3 groups, ANOVA testing was used to determine significant differences. Only *P* values < 0.05 were considered statistically significant.

## SUPPLEMENTARY MATERIAL TABLES



## References

[1] Pietras EM, Warr MR, Passegue E (2011). Cell cycle regulation in hematopoietic stem cells. The Journal of cell biology.

[2] Tesio M, Trumpp A (2011). Breaking the cell cycle of HSCs by p57 and friends. Cell Stem Cell.

[3] Cheng T (2004). Cell cycle inhibitors in normal and tumor stem cells. Oncogene.

[4] Nygren MK, Dosen G, Hystad ME, Stubberud H, Funderud S, Rian E (2007). Wnt3A activates canonical Wnt signalling in acute lymphoblastic leukaemia (ALL) cells and inhibits the proliferation of B-ALL cell lines. British journal of haematology.

[5] Grandori C, Cowley SM, James LP, Eisenman RN (2000). The Myc/Max/Mad network and the transcriptional control of cell behavior. Annual review of cell and developmental biology.

[6] Dang CV (1999). c-Myc target genes involved in cell growth, apoptosis, and metabolism. Mol Cell Biol.

[7] Dang CV, O'Donnell KA, Zeller KI, Nguyen T, Osthus RC, Li F (2006). The c-Myc target gene network. Semin Cancer Biol.

[8] Boxer LM, Dang CV (2001). Translocations involving c-myc and c-myc function. Oncogene.

[9] Liu J, Levens D (2006). Making myc. Curr Top Microbiol Immunol.

[10] Wilson A, Murphy MJ, Oskarsson T, Kaloulis K, Bettess MD, Oser GM, Pasche A-C, Knabenhans C, Macdonald HR, Trumpp A (2004). c-Myc controls the balance between hematopoietic stem cell self-renewal and differentiation. Genes Dev.

[11] Walkley CR, Olsen GH, Dworkin S, Fabb SA, Swann J, McArthur GA, Westmoreland SV, Chambon P, Scadden DT, Purton LE (2007). A microenvironment-induced myeloproliferative syndrome caused by retinoic acid receptor gamma deficiency. Cell.

[12] Raaijmakers MH, Mukherjee S, Guo S, Zhang S, Kobayashi T, Schoonmaker JA, Ebert BL, Al-Shahrour F, Hasserjian RP, Scadden EO, Aung Z, Matza M, Merkenschlager M, Lin C, Rommens JM, Scadden DT (2010). Bone progenitor dysfunction induces myelodysplasia and secondary leukaemia. Nature.

[13] Kode A, Manavalan JS, Mosialou I, Bhagat G, Rathinam CV, Luo N, Khiabanian H, Lee A, Murty VV, Friedman R, Brum A, Park D, Galili N, Mukherjee S, Teruya-Feldstein J, Raza A (2014). Leukaemogenesis induced by an activating beta-catenin mutation in osteoblasts. Nature.

[14] Lutzny G, Kocher T, Schmidt-Supprian M, Rudelius M, Klein-Hitpass L, Finch AJ, Durig J, Wagner M, Haferlach C, Kohlmann A, Schnittger S, Seifert M, Wanninger S, Zaborsky N, Oostendorp R, Ruland J (2013). Protein kinase c-beta-dependent activation of NF-kappaB in stromal cells is indispensable for the survival of chronic lymphocytic leukemia B cells *in vivo*. Cancer cell.

[15] Mendez-Ferrer S, Michurina TV, Ferraro F, Mazloom AR, Macarthur BD, Lira SA, Scadden DT, Ma'ayan A, Enikolopov GN, Frenette PS (2010). Mesenchymal and haematopoietic stem cells form a unique bone marrow niche. Nature.

[16] Calvi LM, Adams GB, Weibrecht KW, Weber JM, Olson DP, Knight MC, Martin RP, Schipani E, Divieti P, Bringhurst FR, Milner LA, Kronenberg HM, Scadden DT (2003). Osteoblastic cells regulate the haematopoietic stem cell niche. Nature.

[17] Zhang J, Niu C, Ye L, Huang H, He X, Tong WG, Ross J, Haug J, Johnson T, Feng JQ, Harris S, Wiedemann LM, Mishina Y, Li L (2003). Identification of the haematopoietic stem cell niche and control of the niche size. Nature.

[18] Brozowski JM, Billard MJ, Tarrant TK (2014). Targeting the molecular and cellular interactions of the bone marrow niche in immunologic disease. Current allergy and asthma reports.

[19] Mendelson A, Frenette PS (2014). Hematopoietic stem cell niche maintenance during homeostasis and regeneration. Nature medicine.

[20] Kunisaki Y, Bruns I, Scheiermann C, Ahmed J, Pinho S, Zhang D, Mizoguchi T, Wei Q, Lucas D, Ito K, Mar JC, Bergman A, Frenette PS (2013). Arteriolar niches maintain haematopoietic stem cell quiescence. Nature.

[21] Wilson A, Oser GM, Jaworski M, Blanco-Bose WE, Laurenti E, Adolphe C, Essers MA, Macdonald HR, Trumpp A (2007). Dormant and self-renewing hematopoietic stem cells and their niches. Ann N Y Acad Sci.

[22] Morrison SJ, Scadden DT (2014). The bone marrow niche for haematopoietic stem cells. Nature.

[23] Lane SW, Scadden DT, Gilliland DG (2009). The leukemic stem cell niche: current concepts and therapeutic opportunities. Blood.

[24] Lo Celso C, Scadden DT (2011). The haematopoietic stem cell niche at a glance. Journal of cell science.

[25] Luo H, Li Q, O'Neal J, Kreisel F, Le Beau MM, Tomasson MH (2005). c-Myc rapidly induces acute myeloid leukemia in mice without evidence of lymphoma-associated antiapoptotic mutations. Blood.

[26] Littlewood TD, Hancock DC, Danielian PS, Parker MG, Evan GI (1995). A modified oestrogen receptor ligand-binding domain as an improved switch for the regulation of heterologous proteins. Nucleic Acids Res.

[27] Pratt WB (1990). Interaction of hsp90 with steroid receptors: organizing some diverse observations and presenting the newest concepts. Molecular and cellular endocrinology.

[28] Bello-Fernandez C, Packham G, Cleveland JL (1993). The ornithine decarboxylase gene is a transcriptional target of c-Myc. Proceedings of the National Academy of Sciences of the United States of America.

[29] Bush A, Mateyak M, Dugan K, Obaya A, Adachi S, Sedivy J, Cole M (1998). c-myc null cells misregulate cad and gadd45 but not other proposed c-Myc targets. Genes & development.

[30] Nakamura Y, Arai F, Iwasaki H, Hosokawa K, Kobayashi I, Gomei Y, Matsumoto Y, Yoshihara H, Suda T (2010). Isolation and characterization of endosteal niche cell populations that regulate hematopoietic stem cells. Blood.

[31] Kamburov A, Stelzl U, Lehrach H, Herwig R (2013). The ConsensusPathDB interaction database: 2013 update. Nucleic acids research.

[32] Sanchez-Aguilera A, Arranz L, Martin-Perez D, Garcia-Garcia A, Stavropoulou V, Kubovcakova L, Isern J, Martin-Salamanca S, Langa X, Skoda RC, Schwaller J, Mendez-Ferrer S (2014). Estrogen signaling selectively induces apoptosis of hematopoietic progenitors and myeloid neoplasms without harming steady-state hematopoiesis. Cell stem cell.

[33] Ding L, Saunders TL, Enikolopov G, Morrison SJ (2012). Endothelial and perivascular cells maintain haematopoietic stem cells. Nature.

[34] Kunisaki Y, Bruns I, Scheiermann C, Ahmed J, Pinho S, Zhang DC, Mizoguchi T, Wei QZ, Lucas D, Ito K, Mar JC, Bergman A, Frenette PS (2013). Arteriolar niches maintain haematopoietic stem cell quiescence. Nature.

[35] Hanahan D, Weinberg RA (2000). The hallmarks of cancer. Cell.

[36] Puca A, Russo G, Giordano A (2012). Properties of mechano-transduction via simulated microgravity and its effects on intracellular trafficking of VEGFR's. Oncotarget.

[37] Holmes K, Roberts OL, Thomas AM, Cross MJ (2007). Vascular endothelial growth factor receptor-2: structure, function, intracellular signalling and therapeutic inhibition. Cellular signalling.

[38] Hall A (1998). Rho GTPases and the actin cytoskeleton. Science (New York, NY).

[39] Etienne-Manneville S, Hall A (2002). Rho GTPases in cell biology. Nature.

[40] Niedergang F, Chavrier P (2005). Regulation of phagocytosis by Rho GTPases. Current topics in microbiology and immunology.

[41] Narumiya S, Yasuda S (2006). Rho GTPases in animal cell mitosis. Current opinion in cell biology.

[42] Istvanffy R, Kroger M, Eckl C, Gitzelmann S, Vilne B, Bock F, Graf S, Schiemann M, Keller UB, Peschel C, Oostendorp RA (2011). Stromal pleiotrophin regulates repopulation behavior of hematopoietic stem cells. Blood.

[43] Miething C, Feihl S, Mugler C, Grundler R, von Bubnoff N, Lordick F, Peschel C, Duyster J (2006). The Bcr-Abl mutations T315I and Y253H do not confer a growth advantage in the absence of imatinib. Leukemia.

[44] Gentleman RC, Carey VJ, Bates DM, Bolstad B, Dettling M, Dudoit S, Ellis B, Gautier L, Ge Y, Gentry J, Hornik K, Hothorn T, Huber W, Iacus S, Irizarry R, Leisch F (2004). Bioconductor: open software development for computational biology and bioinformatics. Genome biology.

[45] Smyth GK (2004). Linear models and empirical bayes methods for assessing differential expression in microarray experiments. Statistical applications in genetics and molecular biology.

[46] Benjamini Y, Hochberg Y (1995). Controlling the False Discovery Rate: A Practical and Powerful Approach to Multiple Testing. Journal of the Royal Statistical Society Series B Methodological.

[47] Kanehisa M, Goto S, Sato Y, Furumichi M, Tanabe M (2012). KEGG for integration and interpretation of large-scale molecular data sets. Nucleic acids research.

[48] Milacic M, Haw R, Rothfels K, Wu G, Croft D, Hermjakob H, D'Eustachio P, Stein L (2012). Annotating cancer variants and anti-cancer therapeutics in reactome. Cancers.

[49] Kelder T, van Iersel MP, Hanspers K, Kutmon M, Conklin BR, Evelo CT, Pico AR (2012). WikiPathways: building research communities on biological pathways. Nucleic acids research.

[50] Kandasamy K, Mohan SS, Raju R, Keerthikumar S, Kumar GS, Venugopal AK, Telikicherla D, Navarro JD, Mathivanan S, Pecquet C, Gollapudi SK, Tattikota SG, Mohan S, Padhukasahasram H, Subbannayya Y, Goel R (2010). NetPath: a public resource of curated signal transduction pathways. Genome biology.

[51] Orchard S, Ammari M, Aranda B, Breuza L, Briganti L, Broackes-Carter F, Campbell NH, Chavali G, Chen C, del-Toro N, Duesbury M, Dumousseau M, Galeota E, Hinz U, Iannuccelli M, Jagannathan S (2014). The MIntAct project--IntAct as a common curation platform for 11 molecular interaction databases. Nucleic acids research.

[52] Chatr-Aryamontri A, Breitkreutz BJ, Oughtred R, Boucher L, Heinicke S, Chen D, Stark C, Breitkreutz A, Kolas N, O'Donnell L, Reguly T, Nixon J, Ramage L, Winter A, Sellam A, Chang C (2015). The BioGRID interaction database: 2015 update. Nucleic acids research.

[53] Keshava Prasad TS, Goel R, Kandasamy K, Keerthikumar S, Kumar S, Mathivanan S, Telikicherla D, Raju R, Shafreen B, Venugopal A, Balakrishnan L, Marimuthu A, Banerjee S, Somanathan DS, Sebastian A, Rani S (2009). Human Protein Reference Database--2009 update. Nucleic acids research.

[54] Ruepp A, Waegele B, Lechner M, Brauner B, Dunger-Kaltenbach I, Fobo G, Frishman G, Montrone C, Mewes HW (2010). CORUM: the comprehensive resource of mammalian protein complexes--2009. Nucleic acids research.

