# Supplementary material for: In vivo hematopoietic Myc activation directs a transcriptional signature in endothelial cells within the bone marrow microenvironment
Source: Oncotarget. 2015 Aug 19;6(26):21827–39. doi: 10.18632/oncotarget.5217 (PMC4673129; doi:10.18632/oncotarget.5217)
Supplement: Supplementary file 1 [file oncotarget-06-21827-s001.pdf]

In vivo hematopoietic Myc activation directs a transcriptional signature in endothelial cells within the bone marrow microenvironment

Supplementary Material

Table S1. Up-regulated DEGs (significant [$p < 0.05$]) differential [log2 fold change (FC) ≥ 1.0] expressed genes) with their symbol, gene denomination, and -fold change between peanut oil (PO) and tamoxifen (Tam) treated samples.

symbol	gene	p.value	Log2 FC
**Tesc**	tescalcin	0.04400	2.57333
**Fam129a**	family with sequence similarity 129, member A	0.04315	2.16667
**Ffar2**	free fatty acid receptor 2	0.00521	1.90667
**Arhgdib**	Rho, GDP dissociation inhibitor (GDI) beta	0.01740	1.88000
**Ncf1**	neutrophil cytosolic factor 1	0.04537	1.86000
**Cotl1**	coactosin-like 1 (Dictyostelium)	0.00878	1.85667
**Pgk1**	phosphoglycerate kinase 1	0.00783	1.82000
**Pygl**	liver glycogen phosphorylase	0.01824	1.75333
**Slfn2**	schlafen 2	0.00490	1.74000
**Mgst2**	microsomal glutathione S-transferase 2	0.02885	1.61000
**Tgfbi**	transforming growth factor, beta induced	0.04219	1.59000
**Cotl1**	coactosin-like 1 (Dictyostelium)	0.00527	1.53667
**Sh3bgrl3**	SH3 domain binding glutamic acid-rich protein-like 3	0.03733	1.52000
**Laptm5**	lysosomal-associated protein transmembrane 5	0.02015	1.40000
**Coro1a**	coronin, actin binding protein 1A	0.03980	1.37333
**2700079J08Rik**	RIKEN cDNA 2700079J08 gene	0.03172	1.32333
**Laptm5**	lysosomal-associated protein transmembrane 5	0.00461	1.27667
**Gfi1**	growth factor independent 1	0.04728	1.26333
**Atp2a3**	ATPase, Ca++ transporting, ubiquitous	0.00725	1.21000
**Pgk1**	phosphoglycerate kinase 1	0.00739	1.17667
**Zfp46**	zinc finger protein 46	0.02393	1.17333
**Ms4a3**	membrane-spanning 4-domains, subfamily A, member 3	0.03504	1.17000
**Fbxo36**	F-box protein 36	0.04160	1.16333
**Tmem51**	transmembrane protein 51	0.04491	1.15667
**Cgrrf1**	cell growth regulator with ring finger domain 1	0.00298	1.13000
**Rac2**	RAS-related C3 botulinum substrate 2	0.04185	1.11667
**Glrx**	glutaredoxin	0.03923	1.11333
**Gpi1**	glucose phosphate isomerase 1	0.01920	1.07333
**Apcdd1**	adenomatosis polyposis coli down-regulated 1	0.02157	1.05667
**Eno1**	enolase 1. alpha non-neuron	0.02763	1.04000
**Ncf2**	neutrophil cytosolic factor 2	0.04989	1.03667
**Tgfbi**	transforming growth factor, beta induced	0.04905	1.02333
**Svs5**	seminal vesicle secretory protein 5	0.01434	1.02000
**Prdx5**	peroxiredoxin 5	0.03071	1.00000

Table S2: Down-regulated DEGs (significant [p < 0.05] differential [log2 fold change (FC) ≤ -1.0] expressed genes) with their symbol, gene denomination, and -fold change between peanut oil (PO) and tamoxifen (Tam) treated samples.

symbol	name	p.value	Log2 FC
**Cyr61**	cysteine rich protein 61	0.03022	-3.44333
**Son**	Son DNA binding protein	0.03417	-3.12333
**2010309G21Rik**	RIKEN cDNA 2010309G21 gene	0.02091	-2.98333
**Cyr61**	cysteine rich protein 61	0.04818	-2.82667
**Chd4**	chromodomain helicase DNA binding protein 4	0.00353	-2.72000
**Kmt2e**	lysine (K)-specific methyltransferase 2E	0.02461	-2.71667
**Ighm**	immunoglobulin heavy constant mu	0.02630	-2.55000
**Dcn**	decorin	0.02404	-2.52333
**Mlec**	malectin	0.03708	-2.49000
**Nktr**	natural killer tumor recognition sequence	0.02509	-2.47667
**Atp2b1**	ATPase, Ca++ transporting, plasma membrane 1	0.00382	-2.35000
**Sptbn1**	spectrin beta, non-erythrocytic 1	0.01909	-2.28000
**Xist**	inactive X specific transcripts	0.00071	-2.25000
**Asph**	aspartate-beta-hydroxylase	0.01158	-2.25000
**G3bp2**	GTPase activating protein (SH3 domain) binding protein 2	0.01646	-2.23667
**Srrm2**	serine/arginine repetitive matrix 2	0.00546	-2.19000
**Brd4**	bromodomain containing 4	0.01224	-2.18000
**Foxp1**	forkhead box P1	0.02885	-2.16000
**Klhl17**	kelch-like 17	0.00920	-2.14667
**Sbno1**	sno, strawberry notch homolog 1 (Drosophila)	0.01937	-2.14667
**Fus**	fused in sarcoma	0.03340	-2.03000
**Diap1**	diaphanous homolog 1 (Drosophila)	0.00060	-1.96333
**Dusp11**	dual specificity phosphatase 11 (RNA/RNP complex 1-interacting)	0.00060	-1.96000
**Csnk2a2**	casein kinase 2. alpha prime polypeptide	0.00269	-1.96000
**Casp3**	caspase 3	0.03293	-1.95333
**Nfix**	nuclear factor I/X	0.01536	-1.94667
**Abcf1**	ATP-binding cassette, sub-family F (GCN20), member 1	0.02804	-1.86667
**Ptp4a2**	protein tyrosine phosphatase 4a2	0.04394	-1.85333
**Stt3b**	STT3. subunit of the oligosaccharyltransferase complex, homolog B (S. cerevisiae)	0.00147	-1.82333
**Prdm2**	PR domain containing 2. with ZNF domain	0.03479	-1.81000
**Malat1**	metastasis associated lung adenocarcinoma transcript 1 (non-coding RNA)	0.00073	-1.80333
**Hsp90ab1**	heat shock protein 90 alpha (cytosolic), class B member 1	0.00300	-1.80000
**Fkbp2**	FK506 binding protein 2	0.02156	-1.80000
**Atp2a2**	ATPase, Ca++ transporting, cardiac muscle, slow twitch 2	0.04075	-1.80000

**1700100M05Rik**	RIKEN cDNA 1700100M05 gene	0.01235	-1.78667
**Celf1**	CUGBP, Elav-like family member 1	0.01975	-1.78667
**Ammecr1l**	AMME chromosomal region gene 1-like	0.01309	-1.77000
**Zzef1**	zinc finger, ZZ-type with EF hand domain 1	0.00775	-1.75000
**Vcp**	valosin containing protein	0.02493	-1.75000
**Scaf4**	SR-related CTD-associated factor 4	0.00752	-1.74000
**Oraov1**	oral cancer overexpressed 1	0.00546	-1.72333
**Nrp2**	neuropilin 2	0.03201	-1.71667
**Evi5**	ecotropic viral integration site 5	0.00616	-1.71000
**C78339**	expressed sequence C78339	0.04843	-1.71000
**AA415437**	expressed sequence AA415437	0.03652	-1.70000
**Lpin2**	lipin 2	0.00571	-1.68667
**Thoc2**	THO complex 2	0.00006	-1.68333
**Tsta3**	tissue specific transplantation antigen P35B	0.04861	-1.67333
**Pisd-ps3**	phosphatidylserine decarboxylase, pseudogene 3	0.04594	-1.66667
**Cp**	ceruloplasmin	0.01610	-1.65667
**H2-M3**	histocompatibility 2. M region locus 3	0.02685	-1.65667
**Srrm1**	serine/arginine repetitive matrix 1	0.00457	-1.64333
**Ermp1**	endoplasmic reticulum metallopeptidase 1	0.01092	-1.64333
**Rab10**	RAB10. member RAS oncogene family	0.00380	-1.61667
**Eif2b2**	eukaryotic translation initiation factor 2B, subunit 2 beta	0.00426	-1.61333
**Lmna**	lamin A	0.03872	-1.60333
**Hsp90aa1**	heat shock protein 90. alpha (cytosolic), class A member 1	0.03162	-1.59667
**Fam104a**	family with sequence similarity 104. member A	0.03173	-1.58000
**Usp10**	ubiquitin specific peptidase 10	0.01414	-1.57333
**Susd2**	sushi domain containing 2	0.03972	-1.56333
**Ehmt1**	euchromatic histone methyltransferase 1	0.02611	-1.55333
**Nfix**	nuclear factor I/X	0.02429	-1.55000
**Arhgef12**	Rho guanine nucleotide exchange factor (GEF) 12	0.00941	-1.53333
**Celf1**	CUGBP, Elav-like family member 1	0.02384	-1.51333
**Snord104**	small nucleolar RNA, C/D box 104	0.00485	-1.50333
**Cbfa2t3**	core-binding factor, runt domain, alpha subunit 2. translocated to, 3 (human)	0.00562	-1.50000
**Tor1aip2**	torsin A interacting protein 2	0.02865	-1.50000
**Ankrd11**	ankyrin repeat domain 11	0.02503	-1.48667
**1200014J11Rik**	RIKEN cDNA 1200014J11 gene	0.02913	-1.48333
**Comt**	catechol-O-methyltransferase	0.04111	-1.48333
**Irf3**	interferon regulatory factor 3	0.04617	-1.47333
**Limd1**	LIM domains containing 1	0.04774	-1.47333
**Rrp36**	ribosomal RNA processing 36 homolog (S. cerevisiae)	0.02007	-1.45000
**Ccar1**	cell division cycle and apoptosis regulator 1	0.03061	-1.45000
**Atxn2**	ataxin 2	0.02865	-1.44333
**Zcchc3**	zinc finger, CCHC domain containing 3	0.02949	-1.43667
**Tmem41b**	transmembrane protein 41B	0.00108	-1.41667

**Snrnp70**	small nuclear ribonucleoprotein 70 (U1)	0.01810	-1.41667
**Map2k2**	mitogen-activated protein kinase kinase 2	0.03163	-1.41333
**Mtdh**	metadherin	0.00649	-1.40667
**Sf3b2**	splicing factor 3b, subunit 2	0.01448	-1.40333
**Trpm7**	transient receptor potential cation channel, subfamily M, member 7	0.03309	-1.40000
**Vegfc**	vascular endothelial growth factor C	0.02911	-1.39667
**Grem1**	gremlin 1	0.04150	-1.39000
**Sdc4**	syndecan 4	0.00401	-1.37333
**BC023829**	cDNA sequence BC023829	0.01503	-1.37333
**Ttc28**	tetratricopeptide repeat domain 28	0.03465	-1.37333
**1600020E01Rik**	RIKEN cDNA 1600020E01 gene	0.04051	-1.37333
**Hectd1**	HECT domain containing 1	0.00662	-1.37000
**Map3k5**	mitogen-activated protein kinase kinase kinase 5	0.03119	-1.35667
**Tmem41b**	transmembrane protein 41B	0.00014	-1.35333
**Dancr**	differentiation antagonizing non-protein coding RNA	0.01597	-1.35333
**Rnf26**	ring finger protein 26	0.04024	-1.35333
**Tor1b**	torsin family 1. member B	0.01521	-1.34000
**Glg1**	golgi apparatus protein 1	0.01951	-1.34000
**Ep400**	E1A binding protein p400	0.04312	-1.32333
**Med13l**	mediator complex subunit 13-like	0.00730	-1.32000
**5430434G16Rik**	RIKEN cDNA 5430434G16 gene	0.04009	-1.30667
**Wipi1**	WD repeat domain, phosphoinositide interacting 1	0.03506	-1.30333
**Lpcat1**	lysophosphatidylcholine acyltransferase 1	0.02074	-1.30000
**Chd4**	chromodomain helicase DNA binding protein 4	0.01293	-1.29667
**Rrp1b**	ribosomal RNA processing 1 homolog B (S. cerevisiae)	0.03321	-1.29333
**Smc6**	structural maintenance of chromosomes 6	0.02523	-1.28667
**Prrc2c**	proline-rich coiled-coil 2C	0.00690	-1.28333
**Cnn3**	calponin 3. acidic	0.01637	-1.28000
**Scarb1**	scavenger receptor class B, member 1	0.02153	-1.27667
**Steap3**	STEAP family member 3	0.02956	-1.27667
**C730029A08Rik**	RIKEN cDNA C730029A08 gene	0.03873	-1.27667
**AI480624**	expressed sequence AI480624	0.01217	-1.27333
**Ppp1r16a**	protein phosphatase 1. regulatory (inhibitor) subunit 16A	0.04064	-1.27333
**Atxn7l3b**	ataxin 7-like 3B	0.03147	-1.27000
**Ncor1**	nuclear receptor co-repressor 1	0.01222	-1.26333
**Gltp**	glycolipid transfer protein	0.01798	-1.26000
**Map4**	microtubule-associated protein 4	0.04048	-1.25667
**Whsc1l1**	Wolf-Hirschhorn syndrome candidate 1-like 1 (human)	0.02880	-1.25333
**Keap1**	kelch-like ECH-associated protein 1	0.04475	-1.25333
**Ralbp1**	ralA binding protein 1	0.01326	-1.25000
**Phf3**	PHD finger protein 3	0.04037	-1.25000
**Atp8a1**	ATPase, aminophospholipid transporter (APLT), class I, type 8A, member 1	0.02148	-1.24667
**Hps1**	Hermansky-Pudlak syndrome 1 homolog (human)	0.03075	-1.24333

**Cryzl1**	crystallin, zeta (quinone reductase)-like 1	0.03363	-1.24000
**Zcrb1**	zinc finger CCHC-type and RNA binding motif 1	0.04278	-1.24000
**Ermp1**	endoplasmic reticulum metallopeptidase 1	0.03136	-1.23333
**Tbl2**	transducin (beta)-like 2	0.04373	-1.23000
**Baz2a**	bromodomain adjacent to zinc finger domain, 2A	0.01985	-1.22667
**Idua**	iduronidase, alpha-L-	0.00868	-1.22333
**Rnf6**	ring finger protein (C3H2C3 type) 6	0.01319	-1.22333
**Scaf4**	SR-related CTD-associated factor 4	0.00006	-1.21333
**Dusp19**	dual specificity phosphatase 19	0.00945	-1.21000
**Vezf1**	vascular endothelial zinc finger 1	0.00666	-1.20667
**Atp13a1**	ATPase type 13A1	0.03119	-1.18667
**Kdm5b**	lysine (K)-specific demethylase 5B	0.02000	-1.18333
**Sp1**	trans-acting transcription factor 1	0.03100	-1.18333
**Bfar**	bifunctional apoptosis regulator	0.03020	-1.18000
**Dpagt1**	dolichyl-phosphate (UDP-N-acetylglucosamine) acetylglucosaminephosphotransferase 1 (GlcNAc-1-P transferase)	0.03196	-1.18000
**Tnrc18**	trinucleotide repeat containing 18	0.01847	-1.17333
**Mrpl38**	mitochondrial ribosomal protein L38	0.00779	-1.17000
**Gpam**	glycerol-3-phosphate acyltransferase, mitochondrial	0.00908	-1.17000
**Rbm25**	RNA binding motif protein 25	0.02906	-1.17000
**Dtx2**	deltex 2 homolog (Drosophila)	0.04380	-1.17000
**Rexo4**	REX4. RNA exonuclease 4 homolog (S. cerevisiae)	0.03339	-1.16333
**6330416G13Rik**	RIKEN cDNA 6330416G13 gene	0.01263	-1.16000
**Ppox**	protoporphyrinogen oxidase	0.04869	-1.15667
**Rnf166**	ring finger protein 166	0.01181	-1.15333
**Atxn7l3b**	ataxin 7-like 3B	0.01754	-1.15333
**Slc19a1**	solute carrier family 19 (folate transporter), member 1	0.04752	-1.14000
**Urgcp**	upregulator of cell proliferation	0.01356	-1.13667
**Mgat1**	mannoside acetylglucosaminyltransferase 1	0.02586	-1.13333
**Dnajc3**	DnaJ (Hsp40) homolog, subfamily C, member 3	0.00031	-1.13000
**Hsp90b1**	heat shock protein 90. beta (Grp94), member 1	0.00039	-1.13000
**Tchp**	trichoplein, keratin filament binding	0.01933	-1.12667
**Chmp7**	charged multivesicular body protein 7	0.02450	-1.12667
**Tmem167**	transmembrane protein 167	0.03701	-1.12667
**Zfp445**	zinc finger protein 445	0.00258	-1.12333
**Synj1**	synaptojanin 1	0.00019	-1.11000
**Rhob**	ras homolog gene family, member B	0.04246	-1.11000
**Psmd11**	proteasome (prosome, macropain) 26S subunit, non-ATPase, 11	0.00123	-1.10333
**Usp40**	ubiquitin specific peptidase 40	0.01008	-1.10333
**Wars**	tryptophanyl-tRNA synthetase	0.03170	-1.10333
**Nedd4**	neural precursor cell expressed, developmentally down-regulated 4	0.03961	-1.09667
**Arhgap19**	Rho GTPase activating protein 19	0.00730	-1.09333

**Ago3**	argonaute RISC catalytic subunit 3	0.02144	-1.09000
**Yeats2**	YEATS domain containing 2	0.04608	-1.08667
**Rab10**	RAB10. member RAS oncogene family	0.00009	-1.08333
**Uckl1**	uridine-cytidine kinase 1-like 1	0.00712	-1.08000
**Nisch**	nischarin	0.03075	-1.08000
**Mettl1**	methyltransferase like 1	0.01822	-1.07667
**Tcf25**	transcription factor 25 (basic helix-loop-helix)	0.01151	-1.07333
**Arih2**	ariadne homolog 2 (Drosophila)	0.01746	-1.07333
**Yy1**	YY1 transcription factor	0.04336	-1.07333
**Fads1**	fatty acid desaturase 1	0.01717	-1.06667
**Polg**	polymerase (DNA directed), gamma	0.00164	-1.06333
**Pigo**	phosphatidylinositol glycan anchor biosynthesis, class O	0.04172	-1.06333
**Luc7l3**	LUC7-like 3 (S. cerevisiae)	0.01453	-1.06000
**Sec62**	SEC62 homolog (S. cerevisiae)	0.01846	-1.05667
**Kdm6a**	lysine (K)-specific demethylase 6A	0.04892	-1.05667
**Ptpn3**	protein tyrosine phosphatase, non-receptor type 3	0.03997	-1.05000
**Fam132a**	family with sequence similarity 132. member A	0.04508	-1.05000
**Larp7**	La ribonucleoprotein domain family, member 7	0.03798	-1.04667
**Ccnl1**	cyclin L1	0.00382	-1.04333
**Mfhas1**	malignant fibrous histiocytoma amplified sequence 1	0.02295	-1.04333
**Masp1**	mannan-binding lectin serine peptidase 1	0.02341	-1.03000
**Pigq**	phosphatidylinositol glycan anchor biosynthesis, class Q	0.02982	-1.03000
**Eif2s2**	eukaryotic translation initiation factor 2. subunit 2 (beta)	0.03403	-1.03000
**Nif3l1**	Ngg1 interacting factor 3-like 1 (S. pombe)	0.02803	-1.02667
**Kmt2c**	lysine (K)-specific methyltransferase 2C	0.03487	-1.02333
**Egln2**	EGL nine homolog 2 (C. elegans)	0.02450	-1.02000
**Hras1**	Harvey rat sarcoma virus oncogene 1	0.01771	-1.01000
**Rnf126**	ring finger protein 126	0.04235	-1.01000
**Zfp692**	zinc finger protein 692	0.00780	-1.00667
**Sass6**	spindle assembly 6 homolog (C. elegans)	0.01174	-1.00667
**LOC100505027**	uncharacterized LOC100505027	0.01456	-1.00667
**Sacm1l**	SAC1 (suppressor of actin mutations 1. homolog)-like (S. cerevisiae)	0.02778	-1.00667
**Lrp10**	low-density lipoprotein receptor-related protein 10	0.03175	-1.00667

Table S3. Enriched pathways.

pathway	p-value	genes of interest within pathway
**Reduction of cytosolic Ca++ levels**	0.00049	ATP2B1;ATP2A2;ATP2A3
**Signaling events mediated by VEGFR1 and VEGFR2**	0.00075	HRAS;NEDD4;HSP90AB1;MAP2K2;VEGFC;HSP90AA1;NRP2
**Hypoxic and oxygen homeostasis regulation of HIF-1-alpha**	0.00157	EGLN2;SP1;ENO1;HSP90AA1;CP;PGK1
**Signaling by GPCR**	0.00200	ARHGEF12;RAC2;HRAS;MAP2K2;HSP90AA1;FFAR2;RHOB
**Platelet calcium homeostasis**	0.00243	ATP2B1;HSP90AA1;MAP2K2;ATP2A2;ATP2A3
**calcium transport I**	0.00263	ATP2A2;ATP2A3
**GPCR downstream signaling**	0.00283	ARHGEF12;RAC2;MAP2K2;HSP90AA1;FFAR2;RHOB
**role of mal in rho-mediated activation of srf**	0.00411	MAP2K2;HRAS;DIAPH1
**Ion transport by P-type ATPases**	0.00453	ATP2B1;ATP8A1;ATP2A2;ATP2A3
**d4gdi signaling pathway**	0.00539	ARHGDIB;CASP3
**Semaphorin interactions**	0.00557	HSP90AB1;RHOB;ARHGEF12;RAC2;HSP90AA1
**Estrogen signaling pathway - Homo sapiens (human)**	0.00619	SP1;HRAS;HSP90B1;HSP90AB1;MAP2K2;HSP90AA1
**Protein processing in endoplasmic reticulum - Homo sapiens (human)**	0.00649	VCP;HSP90B1;DNAJC3;HSP90AB1;SEC62;HSP90AA1;STT3B;MAP3K5
**glycolysis**	0.00695	ENO1;GPI;PGK1
**caspase cascade in apoptosis**	0.00695	LMNA;ARHGDIB;CASP3
**Cross-presentation of particulate exogenous antigens (phagosomes)**	0.00711	NCF2;NCF1
**Sema4D induced cell migration and growth-cone collapse**	0.00780	RHOB;ARHGEF12;RAC2
**gluconeogenesis**	0.00780	ENO1;GPI;PGK1
**RalA downstream regulated genes**	0.00905	RAC2;RALBP1
**RAF/MAP kinase cascade**	0.01119	MAP2K2;HRAS
**VEGF ligand-receptor interactions**	0.01119	NRP2;VEGFC
**VEGF and VEGFR signaling network**	0.01119	NRP2;VEGFC
**hiv-1 nef: negative effector of fas and tnf**	0.01198	LMNA;ARHGDIB;CASP3;MAP3K5

**keratinocyte differentiation**	0.01198	MAP2K2;SP1;HRAS;MAP3K5
**regulation of splicing through sam68**	0.01353	MAP2K2;HRAS
**Signaling by VEGF**	0.01353	NRP2;VEGFC
**Sema4D in semaphorin signaling**	0.01419	RHOB;ARHGEF12;RAC2
**fmlp induced chemokine gene expression in hmc-1 cells**	0.01419	NCF2;HRAS;MAP2K2
**Antigen processing-Cross presentation**	0.01419	NCF2;NCF1;HLA-G
**Signaling by Rho GTPases**	0.01506	ARHGEF12;RAC2;RALBP1;ARHGDIB;RHOB;ARHGAP19
**Rho GTPase cycle**	0.01506	ARHGEF12;RAC2;RALBP1;ARHGDIB;RHOB;ARHGAP19
**mapkinase signaling pathway**	0.01528	MAP2K2;SP1;HRAS;MAP3K5
**Cardiovascular Gene**	0.01555	NCF2;NCF1;FOXP1;YY1;ATP2A2;PRDX5;HLA-G;HSP90B1;MGST2; PPOX;DPAGT1;EIF2B2;NCOR1;ANKRD11;DCN;MGAT1;GPAM;ABCF1; ASPH;MAP3K5;HSP90AB1;ARHGDIB;VEZF1;LPCAT1;EIF2S2;LRP10; IDUA;GREM1;SP1;EVI5;COMT;WARS;FADS1;PIGQ;TBL2;VEGFC; TGFBI;ATP2B1;LMNA;CP;CSNK2A2;NRP2;IRF3;POLG;SBNO1;CASP3; CNN3;MAP2K2;MED13L;SCARB1;SDC4;PIGO;HSP90AA1;PTPN3; SLC19A1
**Prostate cancer - Homo sapiens (human)**	0.01583	HSP90AB1;HSP90AA1;HRAS;HSP90B1;MAP2K2
**L-dopa degradation**	0.01651	COMT
**L-dopa degradation - 11**	0.01651	COMT
**rho cell motility signaling pathway**	0.01681	NCF2;DIAPH1;RALBP1
**EGF-EGFR Signaling Pathway**	0.01739	RALBP1;SP1;HRAS;NEDD4;MAP2K2;SYNJ1;ATXN2
**MEK activation**	0.01877	MAP2K2;HRAS
**RAF phosphorylates MEK**	0.01877	MAP2K2;HRAS
**colanic acid building blocks biosynthesis**	0.01877	TSTA3;GPI
**RAF activation**	0.01877	MAP2K2;HRAS
**SREBP signalling**	0.01910	SP1;SCARB1;GPAM;YY1
**ErbB4 signaling events**	0.01969	NEDD4;NCOR1;CBFA2T3
**HIF-2-alpha transcription factor network**	0.01969	SP1;EGLN2;PGK1
**Amyotrophic lateral sclerosis (ALS)**	0.01969	MAP2K2;CASP3;MAP3K5
**HGF**	0.01969	MAP2K2;HRAS;RAC2
**melanocyte development and pigmentation pathway**	0.02167	MAP2K2;HRAS
**GRB2 events in EGFR signaling**	0.02167	MAP2K2;HRAS
**Oncostatin M Signaling Pathway**	0.02232	MAP2K2;HRAS;CYR61;CASP3
**AGE-RAGE pathway**	0.02346	SP1;NCF1;CASP3;DIAPH1
**CDC42 signaling**	0.02444	NCF2;NCF1;CYR61;DIAPH1;RALBP1;HRAS;ARHGEF12;ARHGDIB

**events**		
**Regulation of CDC42 activity**	0.02444	NCF2;NCF1;CYR61;DIAPH1;RALBP1;HRAS;ARHGEF12;ARHGDIB
**FAS pathway and Stress induction of HSP regulation**	0.02448	LMNA;ARHGDIB;CASP3
**trefoil factors initiate mucosal healing**	0.02448	MAP2K2;HRAS;CASP3
**HIF-1-alpha transcription factor network**	0.02464	SP1;CP;ENO1;PGK1
**Fanconi-bickel syndrome**	0.02473	ENO1;GPI
**SHC1 events in EGFR signaling**	0.02473	MAP2K2;HRAS
**Glycogenosis, Type VII. Tarui disease**	0.02473	ENO1;GPI
**Cori Cycle**	0.02473	GPI;PGK1
**phosphorylation of mek1 by cdk5/p35 down regulates the map kinase pathway**	0.02473	MAP2K2;HRAS
**Sema3A PAK dependent Axon repulsion**	0.02796	HSP90AB1;HSP90AA1
**double stranded rna induced gene expression**	0.02796	EIF2S2;DNAJC3
**Regulation of RAC1 activity**	0.02900	NCF2;ARHGEF12;CYR61;DIAPH1;RALBP1;NCF1;ARHGDIB
**Binding and Uptake of Ligands by Scavenger Receptors**	0.02985	HSP90AA1;SCARB1;HSP90B1
**Regulation of RhoA activity**	0.03055	NCF2;ARHGEF12;CYR61;DIAPH1;RALBP1;NCF1;ARHGDIB
**C-MYC pathway**	0.03120	GPAM;GFI1;SP1;ENO1;HSP90AA1;MTDH
**hypoxia-inducible factor in the cardivascular system**	0.03135	HSP90AA1;ASPH
**MAP kinase cascade**	0.03135	MAP2K2;HRAS
**Bisphosphonate Pathway, Pharmacodynamics**	0.03135	HRAS;RAC2
**cadmium induces dna synthesis and proliferation in macrophages**	0.03135	MAP2K2;HRAS
**human cytomegalovirus and map kinase pathways**	0.03135	SP1;MAP2K2
**N-Glycan biosynthesis**	0.03177	STT3B;DPAGT1;MGAT1
**ascorbate recycling (cytosolic)**	0.03275	GLRX
**GDP-L-fucose biosynthesis I (from GDP-D-mannose)**	0.03275	TSTA3
**RAC1 signaling pathway**	0.03298	NCF2;ARHGEF12;CYR61;DIAPH1;RALBP1;NCF1;ARHGDIB

**SHC-related events triggered by IGF1R**	0.03489	MAP2K2;HRAS
**Mucopolysaccharidosis VI. Sly syndrome**	0.03489	PYGL;GPI
**Glycogenosis, Type III. Cori disease, Debrancher glycogenosis**	0.03489	PYGL;GPI
**Metabolism of folate and pterines**	0.03489	COMT;SLC19A1
**tnfr1 signaling pathway**	0.03489	LMNA;CASP3
**Glycogenosis, Type IV. Amylopectinosis, Anderson disease**	0.03489	PYGL;GPI
**Glycogenosis, Type VI. Hers disease**	0.03489	PYGL;GPI
**Glycogen synthetase deficiency**	0.03489	PYGL;GPI
**Starch and Sucrose Metabolism**	0.03489	PYGL;GPI
**Sucrase-isomaltase deficiency**	0.03489	PYGL;GPI
**Beta3 integrin cell surface interactions**	0.03579	CYR61;SDC4;TGFBI
**Triglyceride Biosynthesis**	0.03579	GPAM;LPIN2;LPCAT1
**GPCR ligand binding**	0.03671	FFAR2;HSP90AA1;MAP2K2
**nfat and hypertrophy of the heart**	0.03789	HRAS;ATP2A2;ATP2A3
**Glycolysis Gluconeogenesis**	0.03789	ENO1;GPI;PGK1
**RAGE**	0.03789	MAP2K2;HRAS;NCF1
**Signaling by constitutively active EGFR**	0.03858	HSP90AA1;HRAS
**RhoA signaling pathway**	0.03914	NCF2;ARHGEF12;CYR61;DIAPH1;RALBP1;NCF1;ARHGDIB
**conversion of glucose to acetyl CoA and entry into the TCA cycle**	0.04006	ENO1;GPI;PGK1
**Axon guidance**	0.04427	ARHGEF12;RAC2;HRAS;HSP90AB1;MAP2K2;HSP90AA1;RHOB; CSNK2A2;NRP2
**HS-GAG degradation**	0.04638	SDC4;IDUA
**Signaling by Leptin**	0.04638	MAP2K2;HRAS
**SOS-mediated signalling**	0.04638	MAP2K2;HRAS
**sprouty regulation of tyrosine kinase signals**	0.04638	MAP2K2;HRAS
**ras signaling pathway**	0.04638	HRAS;RALBP1
**NGF**	0.04638	MAP2K2;HRAS
**SHC1 events in ERBB4 signaling**	0.04638	MAP2K2;HRAS
**Grb2 events in EGFR signaling**	0.04638	MAP2K2;HRAS
**N-Glycan biosynthesis - Homo sapiens (human)**	0.04693	STT3B;DPAGT1;MGAT1

**AndrogenReceptor**	0.04702	CASP3;SP1;HSP90B1;RNF6;HSP90AA1;NCOR1
**Enzymatic degradation of Dopamine by monoamine oxidase**	0.04873	COMT
**Enzymatic degradation of dopamine by COMT**	0.04873	COMT
**dopamine degradation - 11**	0.04873	COMT
**anthrax toxin mechanism of action**	0.04873	MAP2K2
**Synthesis of PIPs at the ER membrane**	0.04873	SACM1L
**glutathione redox reactions II**	0.04873	GLRX
**Chondroitin sulfate/dermatan sulfate metabolism**	0.04934	DCN;SDC4;IDUA
**Developmental Biology**	0.04997	ARHGEF12;RAC2;MED13L;HRAS;HSP90AB1;MAP2K2;NRP2; HSP90AA1;RHOB;CSNK2A2;NCOR1
